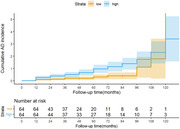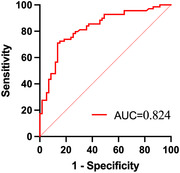# Plasma amyloid‐β Oligomerization tendency as a potential predictor for conversion from mild cognitive impairment to Alzheimer’s dementia: Findings from the GMCII cohort

**DOI:** 10.1002/alz.092632

**Published:** 2025-01-03

**Authors:** Yuhan Xie, Xue Meng, Tao Li, Haifeng Zhang, Yaonan Zheng, SangYun Kim, Chen Zhang, Xin Yu, Huali Wang

**Affiliations:** ^1^ Peking University Institute of Mental Health (Sixth Hospital), Beijing China; ^2^ Beijing Hospital, National Center of Gerontology, Beijing China; ^3^ Seoul National University Bundang Hospital, Seoul National University College of Medicine, Seongnam Korea, Republic of (South); ^4^ Department of Neurology, Tianjin Medical University General Hospital, Beijing China

## Abstract

**Background:**

This study aimed to explore the association between amyloid‐β oligomerization tendency (OAβ) in plasma and cognitive performance in patients with Alzheimer’s disease (AD) and further determine whether plasma OAβ could predict the outcomes of patients with mild cognitive impairment (MCI).

**Method:**

The plasma from 727 subjects in a case registry was tested; these subjects included 286 AD patients, 260 MCI patients and 181 normal controls. The multimer detection system (MDS) was used to measure the plasma oligomeric form of Aβ levels. We measured plasma OAβ after spiking synthetic Aβ into plasma and incubation by this MDS technique. Multipoint clinical diagnosis and domain‐specific cognitive functions were assessed to investigate the relationship between blood biomarkers and clinical AD progression.

**Result:**

Elevated plasma OAβ were strongly correlated with multidomain cognitive performance in patients with MCI and AD. Patients with MCI with high plasma OAβ at baseline demonstrated a higher AD risk (hazard ratio = 1.083, 95% CI 1.032–1.137). Baseline plasma OAβ predicted AD conversion well (AUC = 0.824, 95% CI 0.752–0.897).

**Conclusion:**

Plasma OAβ may be a feasible indicator of AD progression in clinical practice and a potential marker in clinical trials.